
JOURNAL INFORMATION
==============================
NLM Title Abbreviation: Ecol Evol
Journal ID: Ecol Evol
NLM Journal ID: 101566408
Journal ID: ece3

Ecology and Evolution

EISSN: 2045-7758
Publisher: Wiley

ARTICLE INFORMATION
==============================
PMCID: PMC4130439
DOI: 10.1002/ece3.808
Article version: 1
Subjects: Issue Information

Issue Information

Print publication date: 2014 Jul
Electronic publication date: 2014 Jul 21
Volume: 4
Issue: 14
First page: i
Last page: iii
Copyright: © 2014 Published by John Wiley & Sons Ltd.
Copyright year: 2014
License: This is an open access article under the terms of the Creative Commons Attribution License, which permits use, distribution and reproduction in any medium, provided the original work is properly cited.
License URL: https://creativecommons.org/licenses/by/3.0/

==============================
Volume 4, Number 14

### Ecology and Evolution

Open Access

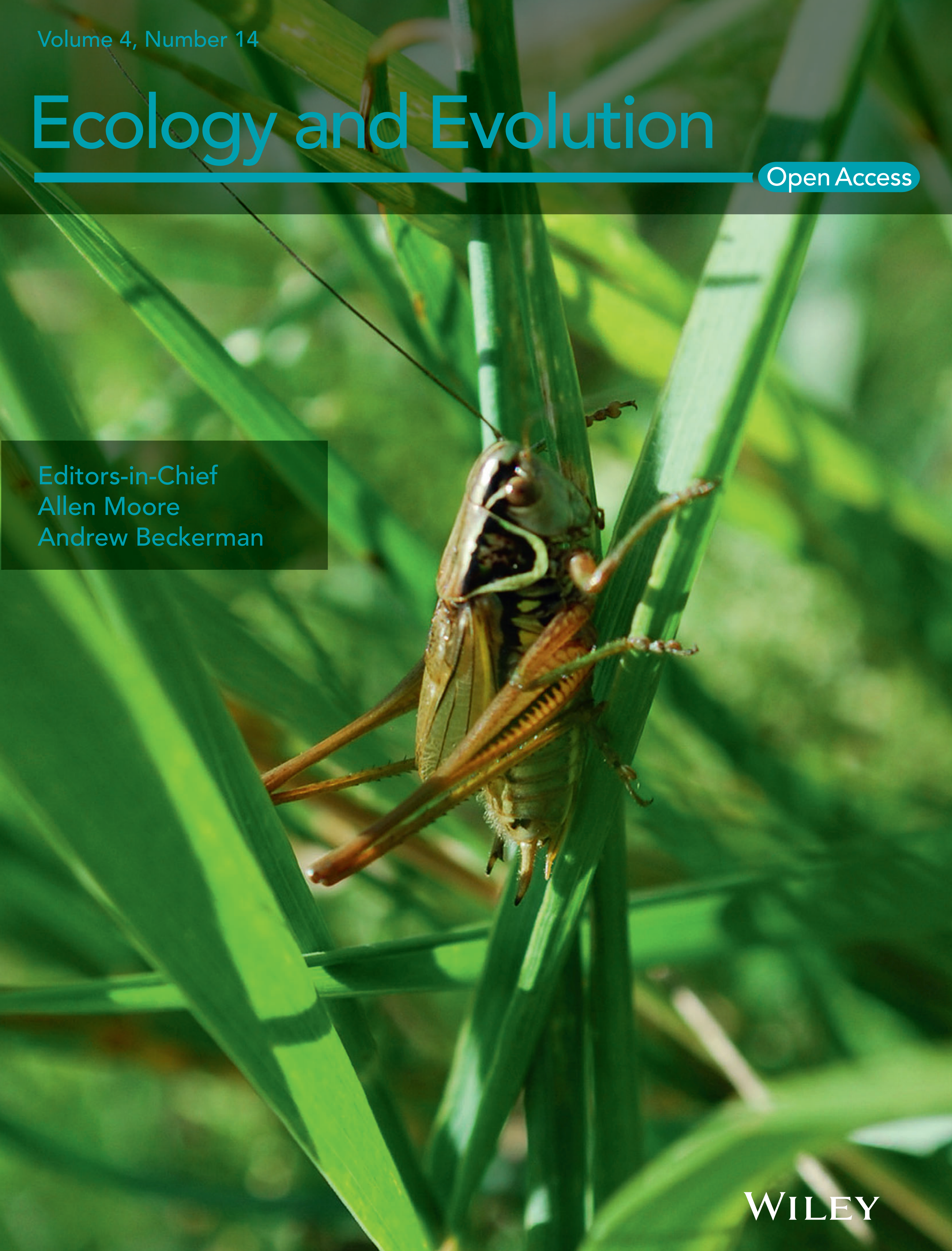

Editors-in-Chief
Allen Moore
Andrew Beckerman

WILEY

Ecology and Evolution

Open Access

### Aims and Scope

*Ecology and Evolution* is the peer-reviewed journal for rapid dissemination of research in all areas of ecology, evolution and conservation science. The journal gives priority to quality research reports, theoretical or empirical, that develop our understanding of organisms and their diversity, interactions between them, and the natural environment.

*Ecology and Evolution* gives prompt and equal consideration to papers reporting theoretical, experimental, applied and descriptive work in terrestrial and aquatic environments. The journal will consider submissions across taxa in areas including but not limited to micro and macro ecological and evolutionary processes, characteristics of and interactions between individuals, populations, communities and the environment, physiological responses to environmental change, population genetics and phylogenetics, relatedness and kin selection, life histories, systematics and taxonomy, conservation genetics, extinction, speciation, adaption, behaviour, biodiversity, species abundance, macroecology, population and ecosystem dynamics, and conservation policy.

*Ecology and Evolution* features original research articles, reviews, editorials, and hypotheses. Original research papers must report well-conducted research with conclusions supported by the data presented in the paper.

*Ecology and Evolution* publishes papers submitted directly to the journal and those referred from a select group of prestigious journals published by Wiley-Blackwell. *Ecology and Evolution* is a Wiley Open Access journal, one of a new series of peer-reviewed titles publishing quality research with speed and efficiency. For further information visit the Wiley Open Access website at http://www.wileyopenaccess.com

### Open Access and Copyright

All articles published by *Ecology and Evolution* are fully open access: immediately freely available to read, download and share. All articles accepted from 14 August 2012 are published under the terms of the Creative Commons Attribution License. All articles accepted before this date were published under a Creative Commons Attribution Non-Commercial License. The Creative Commons Attribution License permits use, distribution and reproduction in any medium, provided the original work is properly cited and allows the commercial use of published articles.

Copyright on any research article in a journal published by *Ecology and Evolution* is retained by the author(s). Authors grant Wiley a license to publish the article and identify itself as the original publisher. Authors also grant any third party the right to use the article freely as long as its integrity is maintained and its original authors, citation details and publisher are identified. Further information about open access license and copyright can be found at http://www.wileyopenaccess.com/details/content/12f25db4c87/Copyright–License.html.

### Purchasing Print Reprints

Print reprints of Wiley Open Access articles can be purchased from corporatesales@wiley.com

### Disclaimer

The Publisher and Editors cannot be held responsible for errors or any consequences arising from the use of information contained in this journal; the views and opinions expressed do not necessarily reflect those of the Publisher and Editors, neither does the publication of advertisements constitute any endorsements by the Publisher and Editors of the products advertised.

Wiley Open Access articles posted to repositories or websites are without warranty from Wiley of any kind, either express or implied, including, but not limited to, warranties of merchantability, fitness for a particular purpose, or non-infringement. To the fullest extent permitted by law Wiley disclaims all liability for any loss or damage arising out of, or in connection with, the use of or inability to use the content.

*Front Cover:* Male Roesel's Bush-cricket (Metrioptera roeselii) on a sunny summer day in Sweden. The picture was taken in Uppland, Sweden.

### Editors-in-Chief

Allen Moore
*Email: allen.moore@ecolevol.org*
Andrew Beckerman
*Email: andrew.beckerman@ecolevol.org*

*Address correspondence to the Editorial Office: ecoevo@wiley.com*

### Editorial Board

Cécile Albert
*McGill University, Canada*

Suzanne H. Alonzo
*Yale University, USA*

Bradley R. Anholt
*University of Victoria, Canada*

Leonardo Bacigalupe
*Universidad Austral de Chile, Chile*

Olga Barbosa
*Universidad Austral de Chile, Chile*

Julia Blanchard
*University of Sheffield, United Kingdom*

Camille Bonneaud
*University of Exeter, United Kingdom*

Mike Boots
*University of Exeter, United Kingdom*

Stan Boutin
*University of Alberta, Canada*

Edmund D. Brodie III
*University of Virginia, USA*

Jarrett Byrnes
*University of Massachucetts, USA*

Martin Dallimer
*University of Leeds, United Kingdom*

Daisy Dent
*University of Stirling, United Kingdom*

Jeremy Draghi
*University of British Columbia, Canada*

John Drake
*University of Georgia, USA*

John Drake
*University of Western Sydney, Australia*

Meghan Duffy
*University of Michigan, USA*

Scott V. Edwards
*Harvard University, USA*

Robert Ekblom
*Uppsala University, Sweden*

Charles B. Fenster
*University of Maryland, USA*

Colin Fontaine
*Muséum de National d'Histoire Naturelle, France*

Vincent Formica
*Swarthmore College, USA*

Charles W. Fox
*University of Kentucky, USA*

Rob Freckleton
*University of Sheffield, United Kingdom*

Brendan Godley
*University of Exeter, United Kingdom*

Patricia Adair Gowaty
*University of California, Los Angeles, USA*

Jessica Gurevitch
*Stonybrook University, USA*

Richard Harrison
*Cornell University, USA*

Stephen Hubbell
*University of California, Los Angeles, USA*

David Johnson
*University of Aberdeen, United Kingdom*

Paula X. Kover
*University of Bath, United Kingdom*

Pavel Kratina
*Queen Mary University of London, United Kingdom*

Mark Ledger
*University of Birmingham, United Kingdom*

Peter Manning
*University of Bern, Switzerland*

Travis Marsico
*Arkansas State University, USA*

Joel McGlothlin
*Virginia Tech, USA*

Mark McPeek
*Dartmouth College, USA*

Alison Nazareno
*Universidade de São Paulo, Brazil*

Roberto Nespolo
*Universidad Austral de Chile, Chile*

Lauren O'Connell
*Harvard University, USA*

Mary O'Connor
*University of British Columbia, Canada*

Arpat Ozgul
*University of Zurich, Switzerland*

Jeff Powell
*University of Western Sydney, Sydney*

Tom Price
*University of Liverpool, United Kingdom*

Catherine Pringle
*University of Georgia, USA*

Laura Reed
*University of Alabama, USA*

Thomas Richards
*Natural History Museum, United Kingdom*

David Shuker
*University of St Andrews, United Kingdom*

Chris Smith
*Earlham College, USA*

Michael Stat
*The University of Western Australia, Australia*

Amber Teacher
*University of Exeter, United Kingdom*

Yit Arn Teh
*University of Aberdeen, United Kingdom*

Gavin Thomas
*University of Sheffield, United Kingdom*

Steven Vamosi
*University of Calgary, Canada*

Mike Whitlock
*University of British Columbia, USA*

Shawn Wilder
*University of Sydney, Australia*

Sam Yeaman
*University of British Columbia, Canada*

Xue-Xian Zhang
*Massey University at Albany, New Zealand*
